# A Review of Journal Impact Metrics and Characteristics to Assist Emergency Medicine Investigators with Manuscript Submission Decisions

**DOI:** 10.5811/westjem.2020.4.47030

**Published:** 2020-07-02

**Authors:** Robert M. Rodriguez, Virginia Chan, Angela H.K. Wong, Juan Carlos C. Montoy

**Affiliations:** University of California, San Francisco, Department of Emergency Medicine, San Francisco, California

## Abstract

**Introduction:**

A crucial, yet subjective and non-evidence-based, decision for researchers is where to submit their original research manuscripts. The approach of submitting to journals in descending order of impact factor (IF) is a common but imperfect strategy. The validity of the IF as a measure of journal quality and significance is suspect, and a number of other journal impact scores have emerged, such that no one scale is universally accepted. Furthermore, practical considerations, such as likelihood of manuscript acceptance rates and times for decisions, may influence how authors prioritize journals. In this report, we sought to 1) review emergency medicine (EM) journal impact metrics, and 2) provide a comprehensive list of pertinent journal characteristics that may influence researchers’ choice of submission.

**Methods:**

We systematically reviewed five impact metrics (IF, H Index, CiteScore, Source-Normalized Impact per Paper, and SCImago Journal Rank) and other relevant characteristics of 20 EM journals.

**Results:**

We found good to excellent agreement in ordinal rankings of four of the journal impact metrics, as measured by the Spearman rank correlation coefficient. The median acceptance rate for original research manuscripts in the EM category was 25% (interquartile range [IQR] 18, 31%), and the median initial decision time was 33 days (IQR 18, 56 days). Fourteen EM journals (70%) accepted brief reports, and 15 (75%) accepted case reports/images.

**Conclusion:**

We recommend replication, expansion, and formalization of this repository of information for EM investigators in a continuously updated, open-access forum sponsored by an independent organization.

## INTRODUCTION

With over 5200 journals currently indexed in Medline,^1^ investigators often a face a daunting task when choosing where to submit their original research manuscripts. The simple *start with the highest impact factor (IF) and work your way down* approach has considerable limitations. Many experts have questioned the validity of the IF as a measure of journal quality and influence.^2^,^3^ Furthermore, a number of other journal impact scores have emerged, such that no one scale is universally accepted as the gold standard impact metric.^4^,^5^

Beyond the limitations of relying on one or more impact metrics, researchers must consider the likelihood of acceptance, time until decision, reach of audience, and expected number of citations. Although the comments to authors after rejections may help improve subsequent submissions, reflexively submitting to high-prestige journals with low likelihood of acceptance can nevertheless waste inordinate amounts of time for decisions and effort toward serial reformatting for particular journal requirements.^6^–^8^ This futile effort can delay investigators from otherwise publishing in less prestigious journals that may be more likely to accept the manuscript, potentially rendering what may have been a timely, novel publication into a stale or redundant article and interrupting the natural evolution of using their published research as a launch point for other projects and grant proposals.

With minimal published guidelines, a common approach for junior (and other) investigators seeking assistance in manuscript submission decisions is to turn to senior academicians for advice – ironically rendering this critical step in their otherwise objective scientific work into a subjective, non-evidence based process. The single, objectively derived decision model for manuscript submissions is one proposed by Wong et al, which requires multiple inputs including journals’ manuscript acceptance rates, times for first decision, and open access fees that may not be readily available.^7^ With the concept of a lack of objective data to assist emergency medicine (EM) investigators with their manuscript submission decisions in mind, we sought to 1) review EM journal impact metrics, and 2) provide a comprehensive list of pertinent journal characteristics that may influence their choice of submission.

## METHODS

### Analysis of Journal Impact Metrics

**Figure 1:**
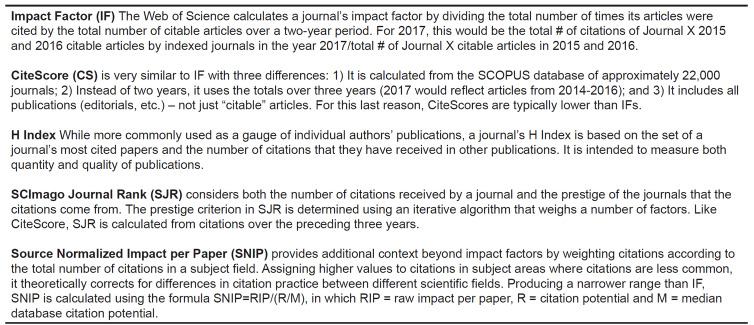
Descriptions of impact metrics.

After review of the most commonly used journal impact metrics,^3^–^5^,^7^–^12^ we analyzed the following journal impact metrics: IF, H index, CiteScore, Source-Normalized Impact per Paper (SNIP), and SCImago Journal Rank (SJR). See Figure 1 for descriptions of these metrics.^8^–^15^ We abstracted values for H index, SNIP, CiteScore, and SJR from websites detailing these factors^11^,^12^ and IF from the Clarivate Analytics 2018 report.^16^ To generate a summary ranking of EM journal impact metrics, we summed each journal’s ordinal rankings according to each of the five impact scores. In this model, the highest impact journal would have the lowest sum of ordinal ranks or the lowest mean ordinal rank.

As a secondary analysis, we sought to compare the agreement of the ordinal rankings of the five IF metrics, ie, the correlation between how the individual metrics ranked journals. For this analysis, we calculated the Spearman rank correlation coefficient, rho, for each pairwise combination of metrics. We conducted these analyses using Stata v13 (StataCorp, College Station, TX) and Excel X for Mac (Microsoft Corporation, Redmond, WA).

### Submission Decision Journal Characteristics

With an explicit goal to provide practical, readily available information to inform submission decisions, we reviewed literature (including the decision model proposed by Wong et al) about pertinent journal characteristics,^7^ and sought to obtain the following features: manuscript acceptance rates; median times for manuscript decision; open access fees/options; and whether journals accept submissions of brief research reports/letters, and case reports/case images. For these characteristics, we first reviewed all of the individual journal official websites. Given that very few published this information, we then sent emails to the contact person(s) listed on these journal websites asking them to provide this data:

1. What is your acceptance rate for original research manuscripts (# accepted for publication/# submitted)?
2. What is your median or mean time for decisions on submitted manuscripts (how many days/weeks/months from submission to time that a decision is rendered and sent to the authors)?

We sent four follow-up emails to non-responders at 10-day intervals and a final inquiry a month after the fourth request. When journals provided vague or incomplete information, the lead investigator asked for further clarification from their editorial staff. As a review of published materials without any patient considerations, this project was categorized as exempt from institutional review board review.

### Journals Reviewed

To generate the journal list, we reviewed the list of top 30 journals in the EM category on the Scimago Journal & Country Rank website (sorted by SJR rank as of May 14, 2019).^11^ We excluded journals with a narrow, non-EM focus, e.g., *Current Heart Failure Reports*, *MicroRNA*, and journals that did not typically publish original research manuscripts (*Emergency Medicine Clinics of North America*). We also excluded journals that did not have a 2017 IF on the 2018 Journal Citation Reports 2018 Clarivate Analytics report of IFs^16^ (the latest version available to us at the time of our analysis) and that did not respond to our queries for their 2017 IF.

## RESULTS

Of the 30 journals listed in the EM category, we excluded eight for irrelevant or narrow focus, one for not publishing original research, and one because of no IF in 2017. We present impact factor metrics and other characteristics of the remaining 20 EM journals in Table 1. Of these 20 journals, 13 (65%) were published out of Europe and seven (35%) were published in the United States. All were English-language journals. Nearly all journals had an open access option with a median charge of $2845. Fourteen journals (70%) accepted brief reports/research letters, and 14 journals (70%) accepted case reports/case images. The median acceptance rate for original research manuscripts was 25% (interquartile range [IQR] 18, 31%) and the median initial decision time was 33 days (IQR 18, 56 days).

**Figure 2:**
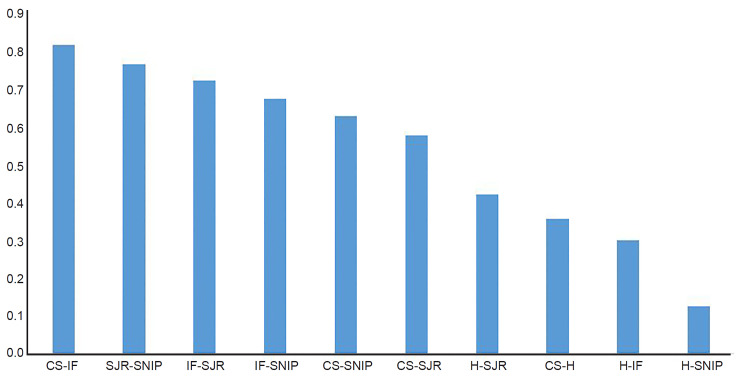
Spearman rank-order correlation. *IF*, Impact factor; *CS*, CiteScore; *SNIP*, Source Normalized Impact per Paper; *SJR*, SCImago Journal Rank; *H*, H Index.

We present the ranking of EM journals by summation of impact factor metrics in Table 2. We calculated the Spearman rank correlation coefficient for each pair of impact metrics; these metrics ranged from 0.13 to 0.82 as presented graphically in Figure 2. The H index showed the lowest agreement with other metrics, and the CiteScore index showed the highest agreement.

## DISCUSSION

Original research investigations are generally laborious and lengthy, often consuming years from start to finish. When considering where to submit the final product of their research for publication, investigators should be afforded as much objective, easily accessible information as possible. Toward this end, we sought to provide a comprehensive review of EM journal impact metrics and other characteristics for investigators.

We found that all but one of the impact metrics showed good to excellent agreement in their ordinal rankings, suggesting that these metrics and their formulas capture only slight nuances in impact. The poor correlation of the H index may be due to the fact that it is generally intended as a metric for authors – not journals. Although several websites provide general descriptions of these and other impact metrics, we were unable to find a similar specific analysis of journal impact metric correlation in any subspecialty field of medicine.

We are not advocating that our summary impact ranking is a general proxy of journal quality, and it should not become a de facto “one-two-three…” template for sequential targeted submission. Detailing all the factors that influence journal choices is beyond the scope of this work. The journal characteristics and the criteria for journal inclusion on these lists were chosen by a single investigator after review of the literature and consideration of the submission decision model proposed by Wong et al. EM investigators and their research are eclectic, and their publication priorities reflect this breadth of experience.^17^ Overall, we recommend that authors use this work to help in their high impact vs likelihood of acceptance computations for submissions. Additionally, although this review is not intended to replace careful inspection of journal websites and instructions for authors, investigators may use our tables as a shortcut to avoid having to slog through numerous websites for other basic journal characteristic information.

Although the information presented in this study is purported to be readily available, we were surprised by the difficult and time-consuming nature of the data collection process. We anticipated that we would only need to conduct simple searches over a month (or less) to gather our desired data – it took nearly five months. Although three websites provided much of the standardized journal impact metric data,^11^,^12^,^16^ they did not offer any of the other journal characteristics we sought to provide. We found information regarding open access options/fees and whether journals accept case reports and brief reports on most journal websites, but it was often buried and sometimes unclear.

Very few journals published information regarding acceptance rates and decision times, and only 28% of our first email inquiries to journal staff were answered. Given these difficulties, we recommend the development of an independently maintained, expanded repository that gathers this information on an ongoing basis – with our work and tables providing a template or roadmap toward this goal. Considerations of conflict of interest or bias toward their affiliated journals notwithstanding, the most logical sponsors of such an endeavor are EM professional organizations such as the Society for Academic Emergency Medicine or the American College of Emergency Physicians. Regardless of who performs this service, the most appropriate home for its output is a freely available, open access website. From a sustainability standpoint, we expect that journals would, over time, recognize the benefits of collaboration and transparency of this repository and provide the input data more freely.

## LIMITATIONS

While journal impact metrics are calculated by third parties in an objective, standardized fashion, the primary limitation of this report is the reliance on journal self-reports for acceptance rates and times for decisions. A few journals either did not respond to our inquiries or stated that they do not provide this information, and so this data remains incomplete. Furthermore, even though we specifically requested data regarding original research, some journals may have provided acceptance rates for *all* types of manuscripts. Similarly, in terms of median/mean times for decisions, their data may have been skewed if they referred to all submissions, including those that were immediately rejected and not sent out for review. Furthermore, use of the median and mean decision times without standard deviations or IQRs of the individual journals may obscure another important factor – the variation in time to decisions within a journal.^6^ Finally, these impact metrics and other journal characteristics are a snapshot of what was available from May–August 2019. Several journals sent us updated IFs and one EM journal that did not have a 2017 IF sent us their newly acquired 2018 IF. To maintain methodologic consistency, we chose not to include updated scores in this report.

## CONCLUSION

We present summary tables of EM journal impact metrics and characteristics to inform original research manuscript submission choices for EM investigators. Considering the effort to acquire this data and annual changes in journal impact metrics, we recommend development of a centralized, open access website repository that can be updated from year to year.

## Figures and Tables

**Table 1:** Emergency medicine journals’ impact factor metrics and journal characteristics (presented alphabetically; N = 20).

Journal*	Country+	IF	H index	CS	SNIP	SJR	Open access fee	Acceptance rate (%)	Decision time** (days)	Brief reports?	Case reports?
Acad Emerg Med	US	2.612	110	2.38	1.352	1.436	$3,000	18	10	Yes	No
Am J Emerg Med	US	1.29	73	1.21	0.746	0.67	$2,550	27	18	Yes	Yes
Ann Emerg Med	US	4.68	137	1.6	1.951	1.439	$3,000	8.3	12	Yes	Yes
CJEM	CA	1.481	39	0.99	0.763	0.456	$3,010	32.4	60	Yes	Yes
Crit Care Resusc	AU	2.014	27	1.52	0.794	1.133	NR	NR	NR	No	Yes
Emerg Med Australas	AU/NZ	1.353	45	0.97	0.725	0.664	$3,300	NR	NR	Yes	Yes
Emerg Med J	UK	2.046	67	1.43	1.216	0.841	£1,950	11	35	Yes	Yes
Euro J Emerg Med	EU	1.729	39	1.11	0.685	0.514	$2,800	12	23	Yes	No
Euro J Trauma Emerg Surg	DE	1.704	18	1.44	0.905	0.45	$3,140	30	44	No	No
Injury	NL	2.199	102	1.99	0.634	0.249	$2,500	NR	56	No	Yes
Intern Emerg Med	IT	2.453	36	1.49	0.709	0.713	$3,760	30	20	Yes	No
J Emerg Med	US	1.207	66	1.04	0.707	0.576	$2,500	23	50	Yes	Yes
Prehosp Disaster Med	UK	0.971	43	0.97	0.671	0.51 1	$1,760	23.2	55	No	Yes
Prehosp Emerg Care	UK	2.269	53	2.45	1.361	1.349	$2,950	21.5	17	Yes	Yes
Resuscitation	NL	5.863	117	3.86	1.944	3.183	$3,000	NR	NR	Yes	No
Scand J Trauma Resusc Emerg Med	NO	2.312	35	2.05	1.251	0.742	$2,325	NR	41	No	Yes
Shock	US	3.005	92	2.6	1.031	1.354	$2,800	25	14	Yes	No
West J Emerg Med	US	1.68#	46	1.65	1.091	0.823	$500	31.3	75	Yes	Yes
Wilderness Environ Med	US	1.161	35	0.87	0.776	0.47	$3,000	38	32	Yes	Yes
World J Emerg Surg	UK	3.198	1.098	3.3	2.137	0.992	$2,890	40	30	No	Yes

* National Library of Medicine Title Abbreviation

+ Country of publication abbreviated according to the United Nations Code List

** Median

# Retrieved from Scimago Journal & Country Rank, not Clarivate (Web of Science, Science Citation Index Expanded).

*IF*, impact factor; *CS*, CiteScore; *SNIP*, Source Normalized Impact per Paper; *SJR*, Scimago Journal & Country Rank; *NR*, no response to queries; $, United States dollars; *OA*, open access.

**Table 2:** Summary ranking (highest to lowest) of top 20 emergency medicine journals by summation of ordinal rankings.

Journal*	IF	H	CS	SNIP	SJR	Average rank	Median rank	Range
Resuscitation	1	2	1	3	1	1.6	1	1 – 3
Ann Emerg Med	2	1	9	2	2	3.2	2	1 – 9
Acad Emerg Med	5	3	5	5	3	4.2	5	3 – 5
Shock	4	5	3	9	4	5	4	3 – 9
Prehosp Emerg Care	8	9	4	4	5	6	5	4 – 9
World J Emerg Surg	3	20	2	1	7	6.6	3	1 – 20
Emerg Med J	10	7	12	7	8	8.8	8	7 – 12
Scand J Trauma Resusc Emerg Med	7	16	6	6	10	9	7	6 – 16
West J Emerg Med	14	10	8	8	9	9.8	9	8 – 14
Intern Emerg Med	6	15	10	16	11	11.6	11	6 – 16
Injury	9	4	7	20	20	12	9	4 – 20
Am J Emerg Med	17	6	13	14	12	12.4	13	6 – 17
Crit Care Med	11	18	19	11	6	13	11	6 – 19
Eur J Emerg Med	13	19	11	10	19	14.4	13	10 – 19
Emerg Med Australas	12	13	14	18	15	14.4	14	12 – 18
Eur J Trauma Emerg S	16	11	17	15	13	14.4	15	11 – 17
J Emerg Med	18	8	15	17	14	14.4	15	8 – 18
CJEM	15	13	16	13	18	15	15	13 – 18
Prehosp Disaster Med	20	12	17	19	16	16.8	17	12 – 20
Wilderness Environ Med	19	16	20	12	17	16.8	17	12 – 20

* National Library of Medicine Title

*IF*, Impact Factor; *CS*, CiteScore; *SNIP*, Source Normalized Impact per Paper; *SJR*, SCImago Journal Rank.
